# RTMS Versus Fluvoxamine in the Treatment of OCD: A Randomized Open-label Pilot Study

**DOI:** 10.7150/ijms.122621

**Published:** 2026-01-01

**Authors:** Yanhua Qin, Liqiang Cai, Lingling Cheng, Ludan Xiang, Xingyue Hu

**Affiliations:** 1 Department of Psychiatry, Sir Run Run Shaw Hospital, Zhejiang University School of Medicine, Hangzhou 310016 Zhejiang, China.; 2 Department of Psychiatry, Tongde Hospital of Zhejiang Province, Hangzhou 310000, Zhejiang, China.; 3 Department of Neurology, Sir Run Run Shaw Hospital, Zhejiang University School of Medicine, Hangzhou 310016, Zhejiang, China.

**Keywords:** obsessive‒compulsive disorder, repetitive transcranial magnetic stimulation, treatment-naïve patients, fluvoxamine

## Abstract

**Background:** Obsessive‒compulsive disorder is a chronic, disabling mental disorder. While repetitive transcranial magnetic stimulation has emerged as a promising neuromodulation intervention for psychiatric disorders, its efficacy in treatment-naïve obsessive‒compulsive disorder patients remains understudied.

**Objective:** This study aimed to test the preliminary efficacy of low-frequency repetitive transcranial magnetic stimulation in treatment-naïve obsessive‒compulsive disorder patients.

**Methods:** Treatment-naïve obsessive‒compulsive disorder patients (n = 41) were randomized to receive either standardized fluvoxamine therapy (150-200 mg/day) or daily low-frequency (1 Hz) repetitive transcranial magnetic stimulation targeting the supplementary motor area for 2 weeks. Clinical outcomes were longitudinally assessed via validated instruments, with a Yale-Brown Obsessive-Compulsive Scale score reduction rate ≥ 25% as the primary endpoint, supplemented by the Beck Depression Inventory and Beck Anxiety Inventory for comorbid symptom evaluation. Safety profiles were monitored throughout the trial.

**Results:** The experimental results revealed that the difference in the response rate at the end of the intervention between the two groups was not statistically significant (***χ^2^*** *= 0.183, p = 0.669*), with 41.7% (5/12) in the repetitive transcranial magnetic stimulation group and 60% (6/10) in the fluvoxamine cohort. No severe adverse events were reported in either group.

**Conclusion:** This trial revealed that low-frequency repetitive transcranial magnetic stimulation over the supplementary motor area might have preliminary positive outcomes for treatment-naïve patients with obsessive‒compulsive disorder. Our findings can be considered a good signal to promote further research in the form of randomized, double-blind, sham-controlled multicenter trials with extended follow-up periods.

## Introduction

Obsessive‒compulsive disorder (OCD) is a chronic, disabling mental disorder characterized by intrusive thoughts or images (obsessions) and repetitive behaviors (compulsions). It may be only obsessive or compulsive symptoms. The lifetime prevalence that fully meets the OCD criteria of the Diagnostic and Statistical Manual of Mental Disorders, fifth edition, is 2.4%, the 12-month prevalence is 1.6% in China 1, and the global prevalence is 1.3% 2. OCD patients and their caregivers have poorer quality of life and greater illness burdens than healthy controls do 3. Selective serotonin-reuptake inhibitors (SSRIs), including fluvoxamine, sertraline, paroxetine and fluoxetine, are recommended as first-line interventions 4; however, 40-60% of patients still exhibit no or little response to these traditional treatments 5. The side effects or low effective rate of medication may sometimes influence obedience to medical orders. Therefore, more alternative options are needed. Deep brain stimulation and gamma knife radiosurgery, alternative treatments for refractory cases, partially improve clinical obsessive and compulsive symptoms. However, both strategies are invasive treatments with side effects, such as brain edema and infection 6-8.

Repetitive transcranial magnetic stimulation (rTMS), a noninvasive treatment with fewer side effects and less time, can change or restore neuron activity via magnetic fields 9. Greenberg first applied rTMS to treat OCD in 1997 10; since then, numerous studies have been conducted in this area. Low-frequency stimulation (≤ 1 Hz) decreases cortical excitability, whereas high-frequency stimulation (≥ 5 Hz) increases underlying cortical excitability 11. Cortical-striatal-thalamo-cortical (CSTC) pathways are related to motor execution control, habit formation, and reward. CSTC pathway activity is strongly associated with OCD symptoms. Inhibitory threshold reduction activates the direct pathway in OCD patients, leading to overactivation of the orbitofrontal cortico-subcutaneous nucleus pathway and making the patient overly concerned with stimuli, such as danger, hygiene, or injury. Patients use compulsive behavior to temporarily alleviate the anxiety and pain caused by the threat 12. According to previous studies, the supplementary motor area (SMA) has extensive connections with subcortical striatum areas involved in response control. Hyperactivity in the SMA may explain deficient inhibitory control over behavior in OCD patients 13. rTMS modulates neural plasticity via long-term depression or potentiation 14. Meta-analysis revealed that the beneficial curative effects of active rTMS are superior to those of the sham group in treating OCD 15, and low-frequency stimulation of the SMA yielded the greatest reductions in Yale-Brown Obsessive-Compulsive Scale (Y-BOCS) scores relative to those of other cortical targets 16. rTMS, as an augmentation, is effective for treating SSRI refractory OCD 17; however, some studies have reported the opposite findings 18. The heterogeneity of participants in clinical trials, including resistant patients, untreated patients, or those under treatment 17, 18, has led to inconsistencies in research findings to some degree. Previous studies have focused primarily on rTMS efficacy in refractory OCD or combined therapy 19-21, making ruling out the possibility of synergism between rTMS and pharmacotherapy difficult. On the basis of the safety of rTMS reported in published articles, no trial has yet been designed to compare rTMS treatment with pharmacotherapy in treatment-free patients directly; therefore, we treated naïve OCD patients with active rTMS or fluvoxamine and compared its effectiveness.

## Methods

### Participants

Eligible participants were males and females recruited from outpatients or inpatients between September 2020 and February 2023. All participants signed an informed consent form prior to inclusion in the study and could comply with the rTMS therapy protocol and scale assessment.

The study inclusion criteria were as follows: (1) met the Diagnostic and Statistical Manual of Mental Disorders, 5th edition criteria for OCD; (2) aged 18--65 years; and (3) were initially diagnosed without medication or other treatment. The exclusion criteria included the following: (1) refractory OCD; (2) any major physical diseases; (3) other severe mental illness illnesses, schizophrenia, current suicidal ideas or attempts (the third item of the Hamilton Depression Rating Scale ≥ 3), bipolar disorder, substance, or alcohol dependence; (4) metallic or foreign implantation in the brain, severe or unstable physical conditions, a history of epilepsy or brain organic diseases, or severe cardiac disorders; and (5) pregnancy, planning to become pregnant, or breastfeeding during the study period.

All study procedures were reviewed and approved by Sir Run Run Shaw Hospital's human research ethics committee (No. 20200908-9).

### Procedures

The participants were assigned to rTMS treatment and fluvoxamine pharmacotherapy groups, with a single random sequence number in a series of opaque and sealed envelopes. We used the CONSORT reporting guidelines during the entire process of the experiment. The demographic data and basic clinical characteristics, including sex, age, age of symptom onset, and duration of disease, were collected. The Y-BOCS is a 10-item scale assessing the severity of OCD symptoms over the past week 22. The Beck Depression Inventory (BDI) is a self-rating scale for assessing depressive symptoms 23, and the Beck Anxiety Inventory (BAI) is a 21-item scale for assessing anxiety symptoms over the past week 24. The BAI and BDI were used to measure the accompanying depressive mood and anxiety, respectively. The clinical raters and rTMS administrators were blinded to the randomization procedure and were separate individuals. The assessment of patient symptoms was performed at baseline and after 2 weeks of treatment via the Y-BOCS, BAI, and BDI. Y-BOCS score reduction rate = (baseline scores - scores at 2 weeks of treatment)/baseline score * 100%. A score reduction rate ≥ 25% was considered an effective response 25. The response rate = (number of effective responses/total number of participants in each group) * 100%.

### Intervention

The rTMS was administered via a 70-mm, eight-shaped coil Magstim Rapid2 stimulator (Magstim Company, Whitland, Wales, United Kingdom) and presented at 100% of the resting motor threshold (RMT) with 1,200 pulses per day for 20 minutes. The participants received 10 treatment sessions, five days a week for either 1 Hz rTMS applied to the SMA. RMT was defined as the minimum TMS intensity required to elicit a motor-evoked potential of the right abductor pollicis brevis muscle in 5/10 trials via single-pulse TMS administered to the left primary motor cortex. The TMS coil was held tangential to the scalp at the stimulation location. The stimulus site was 15% of the distance anterior to the vertex (Cz), corresponding to the bilateral SMA according to the 10-20 International EEG localization system 26.

The dosage of fluvoxamine was started at 50 mg daily and increased slowly within one week to the target dose (150-200 mg daily) with tolerable adverse reactions, after which the dosage was maintained for one week.

### Statistical analyses

All the statistical analyses were performed via R version 4.1.2 software. Finally, 22 samples were collected for statistical analysis. One patient in the rTMS group received seven sessions, but the data were still included in our statistical analysis. Age, illness duration, onset age, and Y-BOCS score are presented as the means ± standard deviations; the differences between the two groups were analyzed via independent sample t tests. The chi-square (*χ^2^*) test or Fischer's exact test was used to compare categorical variables (gender and response rate). The effects of time (baseline and after treatment) and grouping (fluvoxamine and rTMS) variables on secondary outcomes (BDI and BAI) were analyzed via ANCOVA. The alpha level of significance was set at 0.05.

## Results

### Demographic and clinical characteristics at baseline

**Figure 1:**
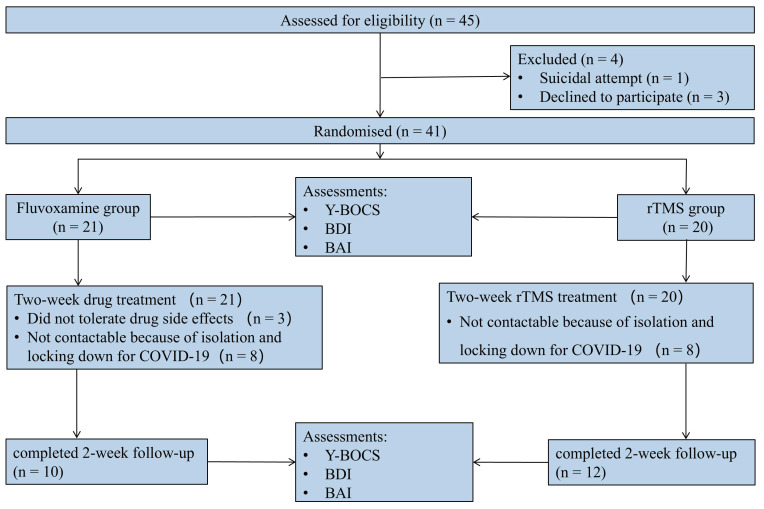
CONSORT diagram of patients with OCD in a randomized open-label clinical study.

A total of 45 eligible participants were males and females recruited from outpatients and inpatients between September 2020 and February 2023. Prior to randomization, 1 patient was excluded from the study because of a suicide attempt, and 3 patients withdrew for personal reasons. The study enrolled 41 patients, 21 and 20 of whom were randomized to the fluvoxamine and rTMS groups, respectively. Among them, 8 patients could not be followed up as planned because of isolation and locking down for COVID-19 in each group, and 3 patients withdrew from the study because of drug side effects. Consequently, data were collected from 22 patients (Figure 1), comprising 10 participants in the drug intervention group and 12 in the rTMS therapy group.

The demographic and clinical characteristics of the study participants are summarized in Table 1. The overall sample consisted of 13 males (59.1%) and 9 females (40.9%). The mean age was 29.08 ± 10.47 years in the rTMS group and 32.4 ± 16.81 years in the fluvoxamine group. The illness duration was 72 ± 90.51 months in the rTMS group and 54 ± 55.16 months in the fluvoxamine group. The baseline Y-BOCS scores were 20 ± 5.13 and 17 ± 5.58 in the rTMS and fluvoxamine groups, respectively. No significant differences were observed between the two groups in terms of sex distribution, illness duration, or baseline morbidity (*p > 0.05*). Similarly, there were no significant differences in the baseline scores for the Y-BOCS, BDI, or BAI (*p > 0.05*).

### Response to the condition during the two weeks of treatment

At the two-week follow-up, 11 out of 22 patients (50%) demonstrated a positive response to treatment (≥ 25% improvement in the Y-BOCS score). Specifically, the response rate for rTMS was 41.7% (5/12), whereas fluvoxamine treatment resulted in a response rate of 60% (6/10). Although the rTMS group had a lower response rate than the fluvoxamine group did, the difference between the two groups was not statistically significant (Table 2) (***χ^2^*** *= 0.183, p = 0.669*, 95% CI = 0.086, 2.628). However, neither depression nor anxiety symptoms improved significantly between the two groups. Repeated measures ANCOVA revealed no significant group effect (*p > 0.05*) or time effect (*p > 0.05*) on BDI/BAI scores before and after treatment (Table 3).

### Adverse events

Among patients receiving rTMS, two of the twelve reported transient headaches following the initial stimulation session, although the pain intensity remained within tolerable limits. Importantly, no subjects withdrew from the rTMS cohort because of adverse events; in contrast, three participants in the fluvoxamine group (3/13, 23.1%) withdrew from the study because severe nausea and dizziness necessitated discontinuation.

## Discussion

### Interpretation

To our knowledge, this is the first investigation of repetitive transcranial magnetic stimulation (rTMS) in treatment-naïve patients with OCD. After 2 weeks of treatment, the response rates were 41.7% (5/12) in the rTMS group and 60% (6/10) in the fluvoxamine group. The therapeutic effect did not differ significantly between the two groups (***χ^2^*** *= 0.183, p > 0.05*), and both groups exhibited similar efficacy. Notably, this pilot study was not powered for equivalence or noninferiority analysis; therefore, we cannot definitively conclude that rTMS is noninferior to fluvoxamine.

Multiple site studies and double-blind trials have demonstrated that modulating the SMA via rTMS could relieve symptoms in patients with resistant OCD. Sarah *et al.* reported that after two weeks of low-frequency rTMS delivered to the SMA in refractory OCD patients, the response rate was 44% (4/9) in the active group and 11% (1/9) in the sham group 27. Our results were consistent with these studies, confirming that inhibiting SMA overactivity could improve OCD symptoms. While prior studies revealed delayed therapeutic onset (6 weeks) with sustained effects posttreatment 26, our cohort, comprising predominantly treatment-naïve and nonrefractory patients, exhibited earlier responsiveness, suggesting differential neuroplasticity in early-stage disease, where these patients might be more responsive to treatment; however, this hypothesis needs to be tested with functional magnetic resonance imaging in the future. While multiple randomized trials have established its efficacy as an augmentation strategy in refractory OCD 18, 26, 28, 29, our findings highlight its viability as a primary intervention in early-stage disease. The therapeutic potential of rTMS extends beyond its monotherapy applications as an adjunctive therapy.

No significant between-group differences emerged in BAI or BDI scores postintervention. This null finding may reflect limited statistical power due to the modest sample size (*n* = 22) and low baseline prevalence of comorbid anxiety/depression in our cohort.

### Limitations

This study has several limitations. First, the design did not include a placebo condition. Given that first-line recommended medications for OCD are widely available and that a greater proportion of patients respond to active low-frequency rTMS delivered to the SMA than to those who receive sham treatment 26, 27, establishing a placebo control group would not align with the best interests of patients. Second, this was an open-label exploratory study with a small sample size. While preliminary positive outcomes were observed, maintenance of efficacy requires longer follow-up, and the underlying neural mechanism needs to be elucidated; thus, larger multicenter trials with double-blind protocols and extended follow-up periods are needed to validate and further investigate the efficacy of rTMS. These trials should additionally employ functional magnetic resonance imaging (fMRI) to verify changes in brain function before and after treatment to validate and further investigate the therapeutic effects of rTMS. Third, the SMA exhibits anatomical variability across individuals. Conventional localization methods based on standardized neuroanatomical landmarks may lead to inaccuracies in targeting stimulation sites because of substantial interindividual variability; owing to the limitations of research funding, we have to depend on these positioning methods. We plan to increase precision by incorporating individualized fMRI-guided navigation in future studies. Fourth, the open-label nature of the trial introduced potential response bias, as the participants were aware of their assigned intervention. Finally, the generalizability of our findings is limited to the specific fluvoxamine (SSRI) and rTMS protocols (1 Hz over the SMA) used in this trial. The results may not apply to other SSRIs, alternative rTMS parameters (e.g., frequency, duration), or different cortical targets. Despite these limitations, our findings provide a meaningful foundation for future research. A randomized, double-blind, sham-controlled trial with longer follow-up is warranted to confirm these results.

## Conclusion

This trial revealed that low-frequency rTMS over SMA might have preliminary positive outcomes for treatment-naïve patients with OCD. Our findings can be considered a good signal to promote further research in the form of randomized, double-blind, sham-controlled multicenter trials.

## Figures and Tables

**Table 1:** Baseline demographic and clinical characteristics of the participants

	Fluvoxamine (n = 10)	rTMS (n = 12)	t/χ^2^	P
Gender (M/F)	5/5	8/4		0.666
Age (years), Mean ± SD	32.4 ± 16.81	29.08 ± 10.47	0.542	0.596
Illness duration (months), Mean ± SD	54 ± 55.16	72 ± 90.5	-0.573	0.574
Onset age (years), Mean ± SD	26.25 ± 12.28	23.08 ± 7.97	0.702	0.494
Y-BOCS score, Mean ± SD	17 ± 5.58	20 ± 5.13	-1.302	0.209
BDI score, Mean ± SD	9.6 ± 7.5	9 ± 5.91	0.205	0.840
BAI score, Mean ± SD	46.4 ± 13.87	41.58 ± 11.52	0.875	0.393

**Table 2:** Response rates in the rTMS and fluvoxamine treatment groups

	Total (n = 22)	Fluvoxamine (n = 10)	rTMS (n = 12)	χ^2^	P	95% CI
Response rate	11 (50%)	6 (60%)	5 (41.7%)	0.183	0.669	(0.086, 2.628)
Nonresponse rate	11 (50%)	4 (40%)	7 (58.3%)			

**Table 3:** BDI and BAI scores at the beginning of treatment and after 2 weeks of treatment

	Baseline (n = 22)			2 weeks of treatment (n=22)			Group		Time
	Fluvoxamine (n = 10)	rTMS (n = 12)		Fluvoxamine (n = 10)	rTMS (n = 12)		F (p)		F (p)
BDI score (Mean ± SD)	9.6 ± 7.5	9 ± 5.9		7.3 ± 7.4	5.9 ± 5.4		0.257 (0.615)		1.958 (0.169)
BAI score (Mean ± SD)	46.4 ± 13.9	41.6±11.5		37.5 ± 13.6	38.8±13.2		0.207 (0.652)		2.050 (0.160)

## Abbreviations

OCD: obsessive‒compulsive disorder
rTMS: repetitive transcranial magnetic stimulation
SMA: supplementary motor area
Y-BOCS: Yale‒Brown Obsessive‒Compulsive Scale
BDI: Beck Depression Inventory
BAI: Beck Anxiety Inventory
SSRIs: selective serotonin-reuptake inhibitors
CSTC: cortical‒striatal‒thalamel-cortical
RMT: resting motor threshold
fMRI: functional magnetic resonance imaging
